# Perioperative temperature management: a survey of 6 Asia–Pacific countries

**DOI:** 10.1186/s12871-021-01414-6

**Published:** 2021-08-16

**Authors:** Wenjun Koh, Murali Chakravarthy, Edgard Simon, Raveenthiran Rasiah, Somrat Charuluxananan, Tae-Yop Kim, Sophia T. H. Chew, Anselm Bräuer, Lian Kah Ti

**Affiliations:** 1 Department, of Anaesthesia, National University Hospital, Singapore, Singapore; 2 Department of Anesthesia, Critical Care and Pain Relief, Fortis Hospital, Bangalore, Karnataka India; 3 Department of Anesthesiology, Philippine General Hospital, University of the Philippines, Ermita, Manila, Philippines; 4 Department of Anesthesiology, Avisena Specialist Hospital, Shah Alam, Selangor Malaysia; 5 Department of Anesthesiology, Faculty of Medicine, Chulalongkorn University, Pathumwan, Bangkok, Thailand; 6 Department of Anesthesiology, Konkuk University Medical Center, Gwangjin-gu, Seoul, Republic of Korea; 7 Department of Anaesthesia, Singapore General Hospital, Singapore, Singapore; 8 Department of Anesthesiology, University Hospital Goettingen, Goettingen, Germany; 9 Department of Anaesthesia, Yong Loo Lin School of Medicine, National University of Singapore, Singapore, Singapore

**Keywords:** Hypothermia, Temperature, Perioperative care, Monitoring, intraoperative, Practice guidelines as topic, Health knowledge, attitudes, practice, Asia

## Abstract

**Background:**

Anesthesia leads to impairments in central and peripheral thermoregulatory responses. Inadvertent perioperative hypothermia is hence a common perioperative complication, and is associated with coagulopathy, increased surgical site infection, delayed drug metabolism, prolonged recovery, and shivering. However, surveys across the world have shown poor compliance to perioperative temperature management guidelines. Therefore, we evaluated the prevalent practices and attitudes to perioperative temperature management in the Asia–Pacific region, and determined the individual and institutional factors that lead to noncompliance.

**Methods:**

A 40-question anonymous online questionnaire was distributed to anesthesiologists and anesthesia trainees in six countries in the Asia–Pacific (Singapore, Malaysia, Philippines, Thailand, India and South Korea). Participants were polled about their current practices in patient warming and temperature measurement across the preoperative, intraoperative and postoperative periods. Questions were also asked regarding various individual and environmental barriers to compliance.

**Results:**

In total, 1154 valid survey responses were obtained and analyzed. 279 (24.2%) of respondents prewarm, 508 (44.0%) perform intraoperative active warming, and 486 (42.1%) perform postoperative active warming in the majority of patients. Additionally, 531 (46.0%) measure temperature preoperatively, 767 (67.5%) measure temperature intraoperatively during general anesthesia, and 953 (82.6%) measure temperature postoperatively in the majority of patients. The availability of active warming devices in the operating room (*p* < 0.001, OR 10.040), absence of financial restriction (*p* < 0.001, OR 2.817), presence of hospital training courses (*p* = 0.011, OR 1.428), and presence of a hospital SOP (*p* < 0.001, OR 1.926) were significantly associated with compliance to intraoperative active warming.

**Conclusions:**

Compliance to international perioperative temperature management guidelines in Asia–Pacific remains poor, especially in small hospitals. Barriers to compliance were limited temperature management equipment, lack of locally-relevant standard operating procedures and training. This may inform international guideline committees on the needs of developing countries, or spur local anesthesiology societies to publish their own national guidelines.

**Supplementary Information:**

The online version contains supplementary material available at 10.1186/s12871-021-01414-6.

## Background

The past few decades have shown an increasing awareness of the physiological mechanisms and effects of temperature on perioperative morbidity and mortality [1]. Inadvertent perioperative hypothermia (IPH) has been defined as a core temperature of < 36 °C in the perioperative period [2].

Anesthesia leads to impairments in central and peripheral thermoregulatory responses. This is exacerbated by cool ambient operating room temperatures and exposed body cavities, resulting in inadvertent perioperative hypothermia in unwarmed surgical patients [3]. Complications include coagulopathy, increased surgical site infection, delayed drug metabolism, prolonged recovery, and shivering [4–6]. Today, temperature monitoring is the standard of care across perioperative monitoring guidelines around the world [7].

In tandem with increasing recognition, an array of options have become available for perioperative patient temperature monitoring and warming. A single layer of passive insulation only compensates for 30% of cutaneous heat losses that occur during general anesthesia, and additional layers of insulation have diminishing effectiveness [3]. Adequate temperature management requires methods of active warming, most commonly forced air warming blankets. Multiple randomized trials [8, 9] and systematic reviews [10–12] have shown the effectiveness of these options in maintaining normothermia, and hospitals have incorporated them into perioperative protocols [1].

Preoperatively, guidelines recommend that the patient’s core temperature be measured before the start of anesthesia, and that elective surgery be postponed until the patient is normothermic [2, 13]. It is also increasingly recognized that prewarming i.e. warming of peripheral tissues before induction of anesthesia [14], is an effective technique to reduce redistributive heat loss intraoperatively, and should optimally be performed for 30 min preoperatively [15–17]. Intraoperatively, most guidelines advise for temperature monitoring when changes in temperature are intended, anticipated or suspected. It is typically recommended that temperature is monitored for patients undergoing general anesthesia for more than 30 min. Guidelines also advocate routine active warming for surgical patients, especially those at higher risk [2, 13]. Postoperatively, temperature monitoring is considered standard of care, and active warming is indicated when patients are hypothermic [2, 7, 18].

Contrary to the growing evidence base surrounding perioperative temperature management, a wave of studies across Europe [19], Australia [20], and China [21] has consistently shown poor compliance to perioperative temperature management guidelines. This study aims to evaluate the prevalent practices and attitudes to perioperative temperature management in the Asia–Pacific region, as well as determine the individual and institutional factors that lead to noncompliance.

## Methods

We conducted a cross-sectional survey on anesthesiologists and anesthesia trainees in six countries in the Asia–Pacific, namely Singapore, Malaysia, Philippines, Thailand, India and South Korea. The survey was conceived in June 2017, and the study protocol was approved by the National Healthcare Group Institutional Review Board (NHG DSRB 2017/00973) prior to study commencement. Written informed consent was waived, and return of anonymous completed questionnaires implied consent to participate. It was then progressively rolled out over an approximately one-and-a-half-year period in the six study countries. All methods were performed in accordance with the relevant guidelines and regulations.

### Survey administration

A 40-question anonymous online questionnaire was developed and distributed via a shareable weblink. This weblink was disseminated to local anesthesiology societies, conferences and hospitals in the surveyed countries. All physicians practising or undergoing training in anesthesiology were invited to participate in the survey. The questionnaires were prefaced by a cover letter describing the survey, and there was no direct contact between study authors and survey participants.

A self-reported questionnaire format was chosen to maximise the outreach of the survey to cover anaesthesia practices from a wide range of settings. This was especially important as at least half of the countries surveyed had a disproportionately large proportion of small hospitals [22], which may be challenging to obtain direct audit data from. The choice of the sharable weblink was to ensure all anaesthesiologists could participate in the survey, as long as they had a valid internet connection and an email address. The authors also felt that the anonymous survey format would encourage more truthful responses as compared to a direct audit, and would hence be more representative of current practices.

To encourage participation and completion of the survey, five vehicle air purifiers were offered as lucky draw prizes for each country. Registration for the lucky draw was optional and conducted with a different form which was linked at the end of the study questionnaire. Participant information from this lucky draw was entirely separate from the study questionnaire, could not be linked back to survey responses in any way, and was not used in the study.

### Questionnaire development

Creation and hosting of the online questionnaire were performed with the web-based survey tool SurveyMonkey [23]. Predominantly closed-ended questions were used, which were a combination of dichotomous, checkbox, multiple select and Likert-scale questions, although options for open-ended responses were provided. Phrases such as “majority of patients” were used when it was recognised that the variable of interest may not be clinically appropriate in all circumstances and patients. Attempts were made to use forced-answer questions where possible, within the limitations of the survey tool, to improve data integrity.

The questionnaire was designed to examine current practices and perceptions, as well as the limitations that may exist that prevent the use and/or adoption of best practices, best monitoring and best interventions for perioperative temperature management. Questions were based on currently published literature as well as the authors’ own experiences, and was jointly constructed and reviewed by authors across the surveyed six Asia–Pacific countries. The primary outcome was to determine the proportions of participants who monitor temperature perioperatively, and actively warm their patients in the preoperative, intraoperative, and postoperative phases. The secondary outcome was to determine the factors that affect compliance to perioperative temperature.

First, the participants’ current practices in patient warming and temperature measurement across the preoperative, intraoperative and postoperative periods were determined. Next, participants were queried regarding the influencing factors and their personal opinions with regards to perioperative temperature management. Finally, participants were asked regarding the availability of patient warming options and temperature measuring equipment in their hospital, as well as any hospital-specific protocols or training courses. To examine the variations across individual practices or countries, additional questions were added to allow for cross-cultural comparison in the exploratory analysis.

### Statistical analysis

Data analysis was conducted using SPSS 23.0 for Windows (IBM, Armonk NY, USA). Descriptive statistics were performed for survey responses and participant demographics. Univariate analyses were performed to identify correlations between demographics and primary variables, and conducted with logistic regression for categorical and ordinal variables, linear regression for continuous variables, and Kruskal–Wallis test for ranked ordinal data.

## Results

A total of 1249 unique survey responses were obtained and exported from the survey software over a one-and-a-half-year period between Oct 2017 to Feb 2019, representing a response rate of 14.9%. Of these responses, 1154 responses (92.4%) were valid. A proportion of questionnaires were largely empty or more than 50% incomplete (7.6%), likely from premature closure of the webpage, and were excluded from the study via case deletion to ensure data integrity. Most respondents practised in India (32.7%), followed by the Philippines (29.6%), Singapore (15.2%) and Malaysia (11.8%). The majority of respondents were specialists (71.6%), and practised in tertiary care hospitals (52.0%). These hospitals range widely in terms of number of beds, number of operating theaters, and number of patients anaesthetized annually. 593 (51.4%) respondents had temperature measuring equipment always available at the operating complex reception or induction room, and 783 (67.9%) respondents had temperature measuring equipment always available at anesthesia recovery area. Similarly, only 521 (45.1%) respondents had active warming devices always available at the operating complex reception or induction room, and 850 (73.7%) respondents had active warming devices always available at the anesthesia recovery area. 624 (59.3%) of respondents were “Often” to “Always” financially restricted in their usage of temperature management equipment. Only 210 (20.0%) respondents’ practice locations conducted training courses on the subject of perioperative temperature management, and 228 (21.7%) had a hospital standard operating procedure (SOP) for perioperative temperature management. Demographic data of the respondents and their practice settings are further elaborated in Table 1.

**Table 1:** Respondents & practice location characteristics

**Characteristic**				**Number**	**Proportion**
Country of practice	India			377	32.7%
	Philippines			342	29.6%
	Singapore			175	15.2%
	Malaysia			136	11.8%
	Thailand			91	7.9%
	South Korea			33	2.9%
Professional designation	Trainee or fellow			328	28.4%
	Specialist			826	71.6%
Hospital type	Primary or Secondary Care			93	8.1%
	Tertiary Care			600	52.0%
	University Hospital			226	19.6%
	Private Hospital			235	20.4%
Number of beds	Less than 250 beds			288	25.0%
	251–500 beds			253	21.9%
	501–1000 beds			355	30.8%
	More than 1000 beds			258	22.4%
Number of operating theaters	Less than 5			221	19.2%
	5 to 10			325	28.2%
	11 to 20			370	32.1%
	More than 20			238	20.6%
Number of patients anesthetized annually	Less than 1000			145	12.6%
	1001 to 10 000			495	42.9%
	10 001 to 20 000			286	24.8%
	More than 20 000			228	19.8%
Locations where temperature measuring equipment is always available	Theater Reception / Induction Room			593	51.4%
	Operating Room			1050	91.0%
	Anesthesia Recovery Area			783	67.9%
Locations where active warming devices are always available	Theater Reception / Induction Room			521	45.1%
	Operating Room			979	84.8%
	Anesthesia Recovery Area			850	73.7%
Financially restricted in temperature management equipment *(n* = *1052)*	Never *139*	Very rarely *71*	Rarely 218	428	40.7%
	Often 357	Very often 128	Always 139	624	59.3%
Presence of hospital training courses *(n* = *1052)*				281	26.7%
Presence of hospital standard operating procedure (SOP) *(n* = *1052)*				228	21.7%

*n* = 1154 for all variables unless otherwise stated

**Fig. 1:**
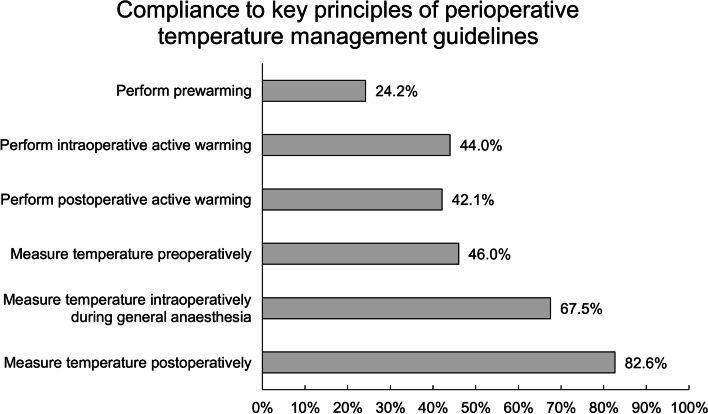
Respondents’ compliance to key principles of perioperative temperature management guidelines

Preoperatively, 531 (46.0%) respondents measure the temperature of the majority of their patients, and 279 (24.2%) respondents perform prewarming for the majority of their patients, and 203 (17.6%) respondents perform prewarming for patients undergoing neuraxial anesthesia. During the intraoperative phase, 767 (67.5%) of respondents measure temperature “Often” to “Always” during general anesthesia, compared to 291 (25.6%) during neuraxial anesthesia. 508 (44.0%) respondents perform intraoperative active warming in the majority of their patients. Postoperatively, 953 (82.6%) of respondents measure temperature in the majority of patients, while 486 (42.1%) respondents perform postoperative active warming for the majority of patients (Table 2). The respondents’ compliance to key principles of perioperative temperature management guidelines are presented in Fig. 1.

**Table 2:** Respondents’ current practices on perioperative temperature management

**Survey question**				**Number**	**Proportion**
**Preoperative phase**					
**Measure temperature preoperatively in the majority of patients**				531	46.0%
**Perform prewarming in the majority of patients**				279	24.2%
Perform prewarming for patients undergoing neuraxial anesthesia				203	17.6%
Duration of prewarming (*n* = *330)*	Less than 10 min			148	44.8%
	10 to 20 min			125	37.9%
	21 to 30 min			36	10.9%
	More than 30 min			21	6.4%
**Intraoperative phase**					
**Measure temperature during general anesthesia *(n* = *1137)***	Never *43*	Very rarely *74*	Rarely *253*	370	32.5%
	Often *363*	Very often *215*	Always *189*	767	67.5%
Measure temperature during neuraxial anesthesia *(n* = *1137)*	Never *242*	Very rarely *192*	Rarely *412*	846	74.4%
	Often *183*	Very often *60*	Always *48*	291	25.6%
Frequency of intraoperative temperature measurement intraoperatively *(n* = *843)*	Continuously			678	80.4%
	Every < 5 min			23	2.7%
	Every 5–10 min			20	2.4%
	Every 10–30 min			50	5.9%
	Every > 30 min			72	8.5%
**Perform intraoperative active warming in the majority of patients**				508	44.0%
Preferred mode(s) of intraoperative cutaneous warming (select all that apply) *(n* = *1017)*	Passive methods (e.g. Blankets)			654	56.7%
	Convection methods (e.g. forced air warmer)			790	68.5%
	Conduction methods (e.g. water mattress)			296	25.6%
	Radiation methods (e.g. infra-red warming devices)			94	10.9%
Average temperature in operating rooms for adult surgery	Less than 21.0 °C			425	36.8%
	21.0—23.0 °C			488	42.3%
	23.1 to 24.0 °C			143	12.4%
	More than 24.0 °C			98	8.5%
**Postoperative phase**					
**Measure temperature postoperatively in the majority of patients**				953	82.6%
Frequency of intraoperative temperature measurement postoperatively *(n* = *443)*	Continuously			83	18.7%
	Every < 5 min			18	4.1%
	Every 5–10 min			32	7.2%
	Every 10–30 min			82	18.5%
	Every > 30 min			228	51.5%
**Perform postoperative active warming in the majority of patients**				486	42.1%

*n* = 1154 for all variables unless otherwise stated. Key perioperative temperature management principles are in bold

On univariate analysis, the availability of active warming devices in the operating room (*p* < 0.001, OR 10.040), absence of financial restriction (*p* < 0.001, OR 2.817), presence of hospital training courses (*p* = 0.011, OR 1.428), and presence of a hospital SOP (*p* < 0.001, OR 1.926) were significantly associated with compliance to intraoperative active warming (Table 3).

**Table 3:** Factors affecting compliance to intraoperative active warming

Variable	Perform intraoperative active warming	Do not perform intraoperative active warming	*p*-value	Odds ratio	95% C.I. for OR	
					Lower	Upper
Active warming devices always available for use in the operating room	492/508 (96.9%)	487/646 (75.4%)	** < 0.001**	10.040	5.915	17.041
“Rarely to never” financially restricted in temperature management equipment ***(n* = *1052)***	271/508 (53.3%)	157/544 (28.9%)	** < 0.001**	2.817	2.183	3.636
Presence of hospital training courses ***(n* = *1052)***	154/508 (30.3%)	127/544 (23.3%)	**0.011**	1.428	1.086	1.879
Presence of hospital standard operating procedure (SOP) ***(n* = *1052)***	139/508 (27.4%)	89/544 (16.4%)	** < 0.001**	1.926	1.427	2.598

*n* = 1154 for all variables unless otherwise stated

When respondents were asked about their perspectives on compliance, a commonly cited barrier to effective perioperative temperature management was the lack of equipment for perioperative temperature monitoring (34.3%), prewarming (34.2%), intraoperative warming (31.6%) and postoperative warming (33.5%). 729 (63.2%) respondents were keen for more active warming devices, and 577 (50.0%) respondents were keen for more temperature measurement devices. Another area which respondents were keen for was more education for staff (73.2%), as well as an implementation of an official hospital standard operating procedure (SOP) (65.2%) (Table 4).

**Table 4:** Respondents’ perspectives on perioperative temperature management

Survey question	Number	Proportion
**Perioperative temperature monitoring**		
I don't believe perioperative temperature monitoring is necessary for the majority of cases	82	7.1%
I am limited by the availability of equipment for perioperative temperature monitoring	396	34.3%
**Prewarming**		
I do not believe prewarming is necessary for the majority of cases	131	11.4%
I am limited by the availability of equipment for prewarming	395	34.2%
There is not enough time to do prewarming	364	31.5%
**Intraoperative warming**		
I do not believe intraoperative warming is necessary for the majority of the cases	15	1.3%
I am limited by the availability of active warming equipment	365	31.6%
I think active warming is not practical as it competes with surgical access	43	3.7%
I think that forced air warmers may increase infection risk by blowing bacteria into the surgical wound	61	5.3%
**Postoperative warming**		
I don't believe postoperative warming is necessary for the majority of the cases	27	2.3%
I am limited by the availability of equipment for postoperative warming	387	33.5%
**Areas that can be improved in the monitoring and prevention of perioperative hypothermia**		
More temperature measurement devices	577	50.0%
Better temperature measurement devices	483	41.9%
More active warming devices	729	63.2%
Better active warming devices	542	47.0%
More education (materials, training) for staff	845	73.2%
Renewing outdated educational materials	374	32.4%
Implementation of an official hospital SOP	752	65.2%
Better enforcement of existing hospital SOP	393	34.1%

*n* = 1154 for all variables unless otherwise stated. Question headings are in bold

Three variables, namely the number of beds, the number of ORs, and the number of patients anesthetized annually, were used to estimate hospital size. As expected, all three variables were highly correlated, and number of ORs was chosen to as the main variable indicative of hospital size as showed the highest correlation to the other study variables.

In the exploratory analysis, it was found that countries differed significantly in terms of the number of operating theaters at the respondent’s practice location (*p* < 0.001). Additionally, an increasing number of operating theaters was significantly associated with the availability of active warming devices (*p* < 0.001) and temperature measurement devices (*p* < 0.001) in the operating room, the absence of financial restriction (*p* < 0.001), the presence of hospital training courses (*p* < 0.001), and presence of a hospital SOP (*p* = 0.001). As the number of operating theaters in their practising location increased, the number of respondents who measure temperature preoperatively (*p* = 0.023), perform prewarming (*p* < 0.001), measure temperature during general anesthesia (*p* < 0.001), perform intraoperative active warming (*p* < 0.001), and perform postoperative active warming (*p* < 0.001) were found to significantly increase (Table 5). Exploratory analyses did not reveal correlations between primary variables and training/professional designation or hospital type.

**Table 5:** Primary variables and participant characteristics grouped by number of operating theaters

Variable	Number of operating theaters				*p*-value	Odds ratio	95% C.I. for OR	
	< 5	5–10	11–20	> 20			Lower	Upper
**Primary variables**								
Measure temperature preoperatively	90 (40.7%)	142 (43.7%)	181 (48.9%)	118 (49.6%)	**0.023**	1.141	1.018	1.279
Perform prewarming	38 (17.2%)	62 (19.1%)	113 (30.5%)	66 (27.7%)	** < 0.001**	1.293	1.130	1.480
Measure temperature intraoperatively during general anesthesia ***(n* = *1137)***	94 (43.7%)	174 (54.4%)	303 (82.6%)	196 (83.4%)	** < 0.001**	2.130	1.856	2.444
Perform intraoperative active warming	58 (26.2%)	122 (37.5%)	197 (53.2%)	131 (55.0%)	** < 0.001**	1.550	1.375	1.746
Measure temperature postoperatively	194 (87.8%)	255 (78.5%)	295 (79.7%)	209 (87.8%)	0.828	1.017	0.876	1.180
Perform postoperative active warming	70 (31.7%)	135 (42.5%)	181 (48.9%)	100 (42.0%)	**0.005**	1.178	1.012	1.370
**Participant characteristics**								
Active warming devices always available for use in the operating room	147 (66.5%)	269 (82.8%)	348 (94.1%)	215 (90.3%)	** < 0.001**	1.999	1.679	2.380
Temperature measurement devices always available for use in the operating room	184 (83.3%)	294 (90.5%)	351 (94.9%)	221 (92.9%)	** < 0.001**	1.516	1.236	1.860
“Rarely to never” financially restricted in temperature management equipment *(n* = *1052)*	184 (83.3%)	294 (90.5%)	351 (94.9%)	221 (92.9%)	** < 0.001**	1.337	1.180	1.515
Presence of hospital training courses *(n* = *1052)*	30 (16.3%)	64 (22.3%)	110 (31.1%)	77 (33.9%)	** < 0.001**	1.389	1.208	1.598
Presence of hospital standard operating procedure (SOP) *(n* = *1052)*	23 (12.5%)	59 (20.6%)	88 (24.9%)	58 (25.6%)	**0.001**	1.292	1.113	1.499

*n* = 1154 for all variables unless otherwise stated

## Discussion

This is the first multinational survey of perioperative temperature management in Asia, and is particularly unique in its inclusion of a large proportion of developing countries. Importantly, a quarter of respondents were from small hospitals with less than 250 beds. A number of international guidelines have been published to reduce inadvertent perioperative hypothermia, largely by national societies based in developed countries [2, 7, 13, 24–29]. None of the studied countries have national guidelines to reduce perioperative hypothermia.

Nevertheless, compliance rates to international perioperative temperature management guidelines across countries and institutions are generally poor [30, 31]. This survey similarly found a poor compliance rate to perioperative temperature management guidelines among respondents. Less than half of respondents (44.0%) perform intraoperative active warming for the majority of their patients. Additionally, less than a quarter of respondents (24.2%) prewarm the majority of their patients. Even when active warming or temperature monitoring is carried out, most respondents do not follow best practices laid out by international guidelines.

The greatest barrier to compliance appears to be the availability of equipment for perioperative temperature management in all three perioperative phases. A substantial proportion of survey respondents do not have ready access to temperature measuring equipment and active warming devices at critical locations, namely the operating complex reception / induction room, the operating theater, and the anesthesia recovery area. Having active warming equipment readily available in the operating room was associated with ten times the odds of performing intraoperative active warming.

Often, the lack of resources is due to financial constraints, which many respondents face. Respondents with financial constraints were about a third as likely to perform intraoperative warming. The association between lack of equipment and noncompliance has also been noted in another national study on perioperative temperature management [32]. It must be emphasized that compliance to guidelines leads to a reduction in perioperative hypothermia and associated adverse events, which can result in net cost savings from fewer complications and a shorter hospital stay [8, 9]. This has been examined in cost analysis reports in the UK [33] and Australia [34].

In the face of significant resource constraints, it can be exceedingly difficult for full compliance to best practices. These guidelines need to be contextualized to the local hospital setting and available resources, such as through hospital training courses or SOPs, to be truly effective. As seen from their survey responses, most respondents already believe in the key tenets of perioperative temperature management guidelines, but are still keen for more training and hospital SOPs on perioperative temperature management. Additionally, respondents in hospitals with training courses or SOPs were 42 and 92% more likely to be compliant to intraoperative active warming respectively. Systematic changes to hospital SOPs have been shown to improve compliance to guidelines and translate into improved clinical outcomes [35–38]. Ideally, various stakeholders in hospital management as well as local experts need to be involved for the conceptualization of the most optimal local strategy, and this can be disseminated into individual hospital training courses or SOPs.

For instance, more than a third of respondents have a cold average operating room temperature of less than 21.0 °C. Raising ambient room temperatures in the induction room and operating theaters can alleviate cutaneous heat losses [39–42]. While this is no replacement for active warming devices, in situations when active warming devices need to be rationed, this can reduce the risk of inadvertent intraoperative hypothermia.

As others have found before [43], it appears that the smaller hospitals face more constraints implementing best practices. Additionally, smaller hospitals also have greater difficulties in terms of resource constraints, and have fewer hospital training courses and hospital SOPs. Having these institutional support mechanisms may be important to improving temperature measurement and patient warming rates in these practice settings. Unfortunately, smaller hospitals also often account for a disproportionately large proportion of patients treated, and this especially true in at least 3 of the 6 countries surveyed [22]. Furthermore, the countries surveyed also tended to have significantly different hospital sizes, which may account for cross-cultural differences in compliance. As these hospitals have the greatest potential for improvement, they should not be neglected in national guidelines and policy-making.

Another significant observation was that compliance rates to intraoperative temperature monitoring during neuraxial anesthesia was half that of general anesthesia (25.6% vs 67.5%), despite the fact that neuraxial anesthesia also impairs thermoregulatory mechanisms to a similar degree as general anesthesia [3]. The importance of intraoperative warming even in patients undergoing neuraxial anesthesia should be further emphasized in subsequent iterations of perioperative temperature management guidelines.

Only a small proportion of respondents feel that active warming is intraoperative warming is unnecessary, or that forced air warming can increase infection risk or interfere with surgical access. While these were traditionally thought to be important barriers to intraoperative active warming, these factors appear to be less important to the study participants, and other factors (eg. resource constraints, training and SOPs) may be more critical.

The focus of this study was to provide a broad overview of perioperative temperature management practices in a wide variety of practice locations. However, as this study was based on self-reported data, there are inherent reporting and recall biases. The study had a relatively limited response rate of 14.9%, which is similar to other published surveys of physicians using a weblink-only survey methodology [44]. Additionally, over 92% of the respondents completed the survey, attesting to the accuracy of the information.

If present, the important sources of response bias would be from respondents who are (1) unable to complete the survey, such as those in low-resource locations without internet access, or (2) are not keen to complete the survey, such as those who do not value perioperative temperature management as important to patient outcomes. These respondents will be under-represented the study. Such biases would be expected to artificially inflate compliance rates, although this was not observed in the study results. Nonetheless, the results of this study should be verified by local audits where possible, ideally in tandem with changes to institutional policies, followed by efforts to close the audit loop.

## Conclusions

In conclusion, this survey found that compliance to perioperative temperature management guidelines is generally poor, especially among smaller hospitals. Environmental/resource limitations is the single largest contributor to noncompliance in the study population as it is a key enabler in effective perioperative temperature management. From an institutional perspective, other areas that are likely to improve compliance rates would be more training on perioperative temperature management, and the development of a hospital SOP. These findings may inform international guideline committees on the needs of developing countries, or may spur local anesthesiology societies to publish their own guidelines specific to the local context.

## Supplementary Information

**Additional file 1.** Asia-Pacific Perioperative Temperature Management Questionnaire. Questionnaire used for data collection.

## Data Availability

The datasets generated and/or analysed during the current study are available in the National University of Singapore Library Database, (URL: https://doi.org/10.25540/8MFS-TT7D).
