# Supplementary material for: Perioperative temperature management: a survey of 6 Asia–Pacific countries
Source: BMC Anesthesiol. 2021 Aug 16;21:205. doi: 10.1186/s12871-021-01414-6 (PMC8365903; doi:10.1186/s12871-021-01414-6)
Supplement: Supplementary file 1 — Additional file 1. Asia-Pacific Perioperative Temperature Management Questionnaire. Questionnaire used for data collection. [file 12871_2021_1414_MOESM1_ESM.docx]

Perioperative temperature management: a survey of 6 Asia-Pacific countries

Wenjun Koh^1^, Murali Chakravarthy^2^, Edgard Simon^3^, Raveenthiran Rasiah^4^, Somrat

Charuluxananan^5^, Tae-Yop Kim^6^, Sophia T.H. Chew^7^, Anselm Bräuer^8^, Lian Kah Ti^1,9*^

^1^ Department of Anaesthesia, National University Hospital, Singapore, Singapore

^2^ Department of Anesthesia, Critical Care and Pain Relief, Fortis Hospital, Bangalore, Karnataka, India

^3^ Department of Anesthesiology, Philippine General Hospital, University of the Philippines, Ermita, Manila, Philippines

^4^ Department of Anesthesiology, Avisena Specialist Hospital, Shah Alam, Selangor, Malaysia

^5^ Department of Anesthesiology, Faculty of Medicine, Chulalongkorn University, Pathumwan, Bangkok, Thailand

^6^ Department of Anesthesiology, Konkuk University Medical Center, Gwangjin-gu, Seoul, Republic of Korea

^7^ Department of Anaesthesia, Singapore General Hospital, Singapore, Singapore

^8^ Department of Anesthesiology, University Hospital Goettingen, Goettingen, Germany

^9^ Department of Anaesthesia, Yong Loo Lin School of Medicine, National University of Singapore, Singapore, Singapore

* Corresponding author

Email: anatilk@nus.edu.sg (LKT)

Asia-Pacific Perioperative Temperature Management Questionnaire

**Demographics**

1. Country of Practice
   1. India
   2. Malaysia
   3. Philippines
   4. Singapore
   5. South Korea
   6. Thailand
   7. Other (please specify)
2. Professional Designation
   1. Trainee/Medical Officer/Resident/Registrar/Fellow
   2. Specialist
3. Hospital Type
   1. Primary/ Secondary Care
   2. Tertiary Care
   3. University Hospital
   4. Private Hospital
4. Number of Beds in Hospital
   1. Less than 250 beds
   2. 251-500 beds
   3. 501-1000 beds
   4. More than 1000 beds
5. Number of Operating Theatres in Hospital
   1. Less than 5
   2. 5 to 10
   3. 11 to 20
   4. More than 20
6. Number of Patients Anaesthetised Annually in Hospital
   1. 1000 or less
   2. 1001 to 10 000
   3. 10 001 to 20 000
   4. More than 20 000

**Operating Room Temperature**

1. What is the estimated average temperature in your hospital’s operating rooms for adult surgery?
   1. Less than 21.0 degrees Celsius
   2. 21.0 - 23.0 degrees Celsius
   3. 23.1 to 24.0 degrees Celsius
   4. More than 24.0 degrees Celsius
2. What is the estimated average temperature in your hospital’s operating rooms for paediatric surgery?
   1. Less than 21.0 degrees Celsius
   2. 21.0 - 23.0 degrees Celsius
   3. 23.1 to 24.0 degrees Celsius
   4. More than 24.0 degrees Celsius
   5. My hospital does not have paediatric patients

**Patient Warming Options at Your Hospital**

1. Are these warming devices available in your hospital?
   1. Blankets (e.g. cotton blankets, space blankets) (Yes/No)
   2. Convective air warming with: (Yes/No)
      1. Upper body blankets
      2. Lower body blankets
      3. Full body blankets
      4. Underbody blankets
      5. Paediatric blankets
      6. Warming gowns
   3. Conductive warmers: (Yes/No)
      1. Electric heating mat under the body
      2. Electric heating mat on top of the body
      3. Water mat under the body
      4. Water mat suit
   4. Other Active Warming Devices: (Yes/No)
      1. Radiation (Infra-red) warmers
      2. Fluid Infusion warmers
      3. Electric or powered humidifiers
   5. Other (please specify)
2. Does your hospital have the necessary equipment to warm surgical irrigation fluids? (e.g. for cases such as Transurethral Resection of the Prostate (TURP)?)
   1. Yes
   2. No
3. In your hospital, which of the following anaesthesia work areas or locations is temperature measuring equipment always available? (Select ALL that apply)
   1. Operating Complex Reception/Waiting Area
   2. Anaesthesia Induction Room
   3. Operating Room
   4. Obstetric Delivery Room
   5. Radiology/ Cardiology Diagnostic or Intervention Areas
   6. Anaesthesia Recovery Area
   7. None of the Above
4. In your hospital, which of the following anaesthesia work areas or locations are active warming devices always available? (Select ALL that apply)
   1. Operating Complex Reception/Waiting Area
   2. Anaesthesia Induction Room
   3. Operating Room
   4. Obstetric Delivery Room
   5. Radiology/ Cardiology Diagnostic or Intervention Areas
   6. Anaesthesia Recovery Area
   7. None of the Above

**Measurement of Core Body Temperature - by Anaesthetic Technique**

1. Do you measure your patient's Core Body Temperature during:
   1. General Anaesthesia?
      1. Never
      2. Very Rarely
      3. Rarely
      4. Often
      5. Very Often
      6. Always
   2. Spinal/ Epidural Anaesthesia?
      1. Never
      2. Very Rarely
      3. Rarely
      4. Often
      5. Very Often
      6. Always
   3. Peripheral Nerve Block?
      1. Never
      2. Very Rarely
      3. Rarely
      4. Often
      5. Very Often
      6. Always

**Preoperative Period**

1. Do you measure core temperature of your patients preoperatively at least 50% of the time? (Select ALL that apply):
   1. No - please skip to Q16.
   2. Yes, in the ward before patient is called to the operating complex
   3. Yes, in the operating complex reception/ waiting area
   4. Yes, in the anaesthesia induction room
2. Which route do you use to measure core body temperature before Anaesthesia Induction? (Please select Most commonly used and Second most commonly used. If there is no "2nd most common method", choose "Other", and specify "NA".)
   1. Oral/Sublingual
   2. Tympanic membrane
   3. Other (please specify in the text box below)
3. Do you perform prewarming before initiation of anaesthesia in at least 50% of your patients?
   1. Yes
   2. No – please skip to Q17

16a. If yes, in which location do you usually perform prewarming?

- 1. In operating complex reception/waiting area
  2. In anaesthesia induction room
  3. Other (please specify)

16b. How long do you prewarm your patients?

- 1. Less than 10 minutes
  2. 10 to 20 minutes
  3. 21 to 30 minutes
  4. More than 30 minutes

1. Do you perform prewarming for patients undergoing epidural or spinal anaesthesia?
   1. Yes
   2. No

**Intraoperative Period**

1. Which route do you use to measure core body temperature intraoperatively? (Please select Most commonly used and Second most commonly used. If there is no "2nd most common method", choose "Other", and specify "NA".)
   1. None/ Not Applicable
   2. Nasopharynx
   3. Oral/Sublingual
   4. Bladder
   5. Rectum
   6. Tympanic membrane
   7. Other (please specify in the text box below)
2. At what time intervals do you measure core body temperature intraoperatively?
   1. I do not measure
   2. Intermittently
   3. Continuously
   4. Every 1 to 5 minutes
   5. Every 6 to 10 minutes
   6. Every 11 to 29 minutes
   7. Every 30 minutes or longer
3. My preferred mode(s) of intraoperative warming is/are: (Select ALL that apply)
   1. I do not usually use intraoperative warming
   2. Passive/Preventative methods (e.g. blankets, heat & moisture exchanger)
   3. Convection methods (e.g. forced air warmer)
   4. Conduction methods (e.g. water mattress)
   5. Radiation methods (e.g. infra-red warming devices)
   6. Warmed fluids
   7. Fluid infusion warmer (e.g. inline warming devices)

20a. If you chose fluid infusion warming in the question above (i.e. Q20), what is your expected fluid throughput indication?

- 1. Greater than 1000ml/h
  2. Greater than 500ml/h
  3. Regardless of expected fluid throughput

**Postoperative Period - Post-Anaesthesia Care Unit (PACU) or Anaesthesia Recovery Area**

1. Which route do you use to measure core body temperature postoperatively?
   1. None/ Not Applicable
   2. Nasopharynx
   3. Oral/Sublingual
   4. Bladder
   5. Rectum
   6. Tympanic membrane
   7. Other (please specify in the text box below)
2. At what time intervals do you measure core body temperature postoperatively?
   1. I do not measure
   2. Intermittently
   3. Continuously
   4. Every 1 to 5 minutes
   5. Every 6 to 10 minutes
   6. Every 11 to 29 minutes
   7. Every 30 minutes or longer
3. My preferred mode(s) of postoperative warming is/are: (Select ALL that apply)
   1. I do not usually use intraoperative warming
   2. Passive/Preventative warming (e.g. blankets, heat & moisture exchanger)
   3. Convection methods (e.g. forced air warmer)
   4. Conduction methods (e.g. water mattress)
   5. Radiation methods (e.g. infra-red warming devices)
4. At what body core temperature would you NOT extubate patients, and move ventilated patient into the recovery room or into the ICU?
   1. Body Temperature is not a criterion for extubation in my practice
   2. Less than 34 degrees Celsius
   3. Less than 35 degrees Celsius
   4. Less than 36 degrees Celsius
5. How do you treat postoperative shivering? (Select ALL that apply):
   1. Passive warming (e.g. blankets)
   2. Active warming devices
   3. Administration of Clonidine
   4. Administration of Pethidine
   5. Other (please specify)

**Standards and Protocols at Your Hospital**

1. Does your hospital have an official standard operating procedure (SOP) or protocol for patient perioperative temperature management?
   1. Yes
   2. No - please skip to Q27

26a. If yes, what is included in the SOP/protocol? (Select ALL that apply):

- 1. Advocacy of site and/or device for temperature measurement
  2. Requirement for temperature measurement before anaesthesia introduction
  3. Prewarming before anaesthesia introduction
  4. Guidelines on intraoperative warming strategies
  5. Guidelines for extubation of hypothermic patients
  6. Postoperative warming for hypothermic patients
  7. Providing patients with information about the risks of perioperative hypothermia
  8. Checklist for perioperative temperature management

1. Does your hospital conduct training courses on the subject of perioperative hypothermia?
   1. Yes
   2. No
2. Does your hospital conduct training courses on the use of materials or equipment for the monitoring and prevention of perioperative hypothermia?
   1. Yes
   2. No
3. In your opinion, what are the areas that can be improved in the monitoring and prevention of perioperative hypothermia? (Select ALL that apply):
   1. More education (materials, training) for staff
   2. Renewing outdated educational materials
   3. Implementation of an official hospital SOP or protocol
   4. Better enforcement of existing hospital SOP or protocol
   5. More temperature measurement devices
   6. Better temperature measurement devices
   7. More active warming devices
   8. Better active warming devices
4. In your practice, are you financially restricted with the choice or use of perioperative monitoring and warming devices?
   1. Never
   2. Very Rarely
   3. Rarely
   4. Often
   5. Very often
   6. Always

**Your Individual Practice for Temperature Management** - For each of the following scenarios/situations/indications, please choose ALL options that you would routinely do (i.e. more than 50% of the time) in your usual practice

1. Laparoscopic or Thoracoscopic Surgery
   1. Monitor temperature of the patient
   2. Prewarm patient
   3. Utilise active warming intraoperatively
   4. Perform active warming postoperatively in PACU or recovery area
   5. None of the above
2. Open Cavity Surgery (e.g. Laparotomy, Thoracotomy)
   1. Monitor temperature of the patient
   2. Prewarm patient
   3. Utilise active warming intraoperatively
   4. Perform active warming postoperatively in PACU or recovery area
   5. None of the above
3. Expected Length of Surgery:
   1. I consider length of surgery as an indication to monitor and/or actively warm patients.
      1. Yes
      2. No
   2. For surgeries of short duration of less than 1 hour:
      1. No routine temperature monitoring or warming intervention
      2. Patient temperature monitoring
      3. Patient prewarming
      4. Active warming intraoperatively
      5. Active warming postoperatively in PACU or recovery area
   3. For surgeries of moderate duration of 1 to 3 hours:
      1. No routine temperature monitoring or warming intervention
      2. Patient temperature monitoring
      3. Patient prewarming
      4. Active warming intraoperatively
      5. Active warming postoperatively in PACU or recovery area
   4. For surgeries of long duration of more than 3 hours:
      1. No routine temperature monitoring or warming intervention
      2. Patient temperature monitoring
      3. Patient prewarming
      4. Active warming intraoperatively
      5. Active warming postoperatively in PACU or recovery area
4. Estimated Blood Loss:
   1. I consider estimated blood loss as an indication to monitor and/or actively warm patients.
      1. Yes
      2. No
   2. For surgeries in which the estimated blood loss is minimal (i.e. <10% of estimated blood volume or <500ml):
      1. No routine temperature monitoring or warming intervention
      2. Patient temperature monitoring
      3. Patient prewarming
      4. Active warming intraoperatively
      5. Active warming postoperatively in PACU or recovery area
   3. For surgeries in which the estimated blood loss is moderate (i.e. 10-20% of estimated blood volume or 500-1000ml):
      1. No routine temperature monitoring or warming intervention
      2. Patient temperature monitoring
      3. Patient prewarming
      4. Active warming intraoperatively
      5. Active warming postoperatively in PACU or recovery area
   4. For surgeries in which the estimated blood loss is severe (i.e. >20% of estimated blood volume or >1000ml):
      1. No routine temperature monitoring or warming intervention
      2. Patient temperature monitoring
      3. Patient prewarming
      4. Active warming intraoperatively
      5. Active warming postoperatively in PACU or recovery area
5. Age of Patient:
   1. I consider age as an indication to monitor and/or actively warm patients.
      1. Yes
      2. No
   2. For paediatric patients (Less than age 12):
      1. No routine temperature monitoring or warming intervention
      2. Patient temperature monitoring
      3. Patient prewarming
      4. Active warming intraoperatively
      5. Active warming postoperatively in PACU or recovery area
   3. For geriatric patients (More than age 65):
      1. No routine temperature monitoring or warming intervention
      2. Patient temperature monitoring
      3. Patient prewarming
      4. Active warming intraoperatively
      5. Active warming postoperatively in PACU or recovery area

**Your Individual Practice: Influencing Factors & Personal Opinions** - For the following questions, please select ALL that apply.

1. Monitoring temperature for patients undergoing anaesthesia (GA, Spinal/Epidural, Peripheral Nerve Blocks):
   1. I already monitor most of my patients
   2. I would like to monitor more of my patients
   3. I am limited by the availability of equipment
   4. I am concerned about the cost of monitoring
   5. I don't believe monitoring is necessary for the majority of the cases
2. Prewarming patients:
   1. I already do prewarming for most of my patients
   2. I would like to do prewarming for more of my patients
   3. I am limited by the availability of equipment
   4. There is not enough time to do prewarming
   5. I don't believe prewarming is necessary for the majority of the cases
3. Active vs. Passive warming devices
   1. I use passive warming devices in the majority of my patients
   2. I use active warming devices in the majority of my patients
   3. I believe that passive warming devices alone are adequate in the majority of patients
   4. I believe that active warming devices are needed for the majority of patients
   5. I think that active warming devices provide value for money
4. Intraoperative warming
   1. I use passive warming devices in the majority of my patients
   2. I use active warming devices in the majority of my patients
   3. I use active warming devices for selected cases only
   4. I would like more of my patients to receive active warming
   5. I am limited by the availability of active warming equipment
   6. I am concerned about the cost of active warming
   7. I think active warming is not practical as it competes with surgical access
   8. I think that forced air warmers may increase infection risk by blowing bacteria into the surgical wound
   9. I don't believe intraoperative warming is necessary for the majority of the cases
5. Postoperative warming in the PACU or Anaesthesia Recovery Area
   1. I already use postoperative warming for most of my patients
   2. I would like more of my patients to receive postoperative warming
   3. I am limited by the availability of equipment
   4. I am concerned about the cost of postoperative warming
   5. I don't believe postoperative warming is necessary for the majority of the cases
